# Characterization of Klebsiella Phages Isolated Against a Clinical Host with High Genome and Proteome Identity but Variable Tail Fibers

**DOI:** 10.3390/v18040430

**Published:** 2026-04-01

**Authors:** Jessica M. Lewis, Daniel K. Arens, Nathan R. Zuniga, Julianne H. Grose

**Affiliations:** 1 Department of Microbiology and Molecular Biology, Brigham Young University, Provo, UT 84040, USA; jessica.lewis@byu.edu (J.M.L.); danielarens09@gmail.com (D.K.A.); 2 Department of Chemistry, Brigham Young University, Provo, UT 84040, USA; natezu93@gmail.com

**Keywords:** *Klebsiella* phage, clinical, *Jiaodavirus*, T4-like

## Abstract

The rate at which bacteria are gaining resistance to antibiotics is outpacing the discovery of new drugs. The rise of superbugs such as Carbapenem-resistant and Extended-Spectrum Beta-Lactamase Producing *Enterobacteriaceae* are leading to infections that are resistant to our last lines of defense. One of the most prolific genera of these bacteria is *Klebsiella*, which causes one third of Gram-negative infections. The need for alternative and companion treatments has never been greater. Bacteriophages are bacteria-infecting viruses with high specificity to their host. They show great promise as a potential treatment for antibiotic-resistant infections. Here, we describe the characterization of five closely related bacteriophages (ValerieMcCarty01–05) isolated against an antibiotic-resistant clinical strain of *Klebsiella oxytoca*, which is an emerging antimicrobial-resistant threat within the *Klebsiella* genus. These phages demonstrate high similarity at both the genomic and proteomic levels and share homology with other T4-like *Enterobacterales* phage. Two phages were further characterized through a mass spectrometry analysis of purified virions, identifying peptide spectrum matches for 40 proteins which appear to be virion proteins. In addition, the peptide spectrum matches for 39 hypothetical proteins suggest they are indeed proteins. Amino acid alignment revealed that the tail fibers display more variability than most of their genome, suggesting possible adaptive tail fiber gene shuffling. Despite this variability, these phages maintained broad but high specificity for *Klebsiella* species in this paper, including *K. oxytoca*, *K. pneumoniae* and *K. aerogenes* and several clinical *Klebsiella* isolates, with infectivity differences seen only in efficiency. This specificity for *Klebsiella* is consistent with the genus to which they belong (the *Jiaodavirus*, which contains only *Klebsiella* phages) and suggests they may be involved in the evolution of *Klebsiella* and be useful therapeutics.

## 1. Introduction

The discovery of penicillin in the 1920s, followed by its widespread use in the 1940s, is arguably one of the most significant milestones in the history of medical science. This breakthrough ushered in a period of rapid antibiotic discovery from the 1950s through the 1970s. Since then, however, the development of new antibiotic classes has slowed dramatically, which is largely due to the substantial time and financial investment required to evaluate the safety and specificity of novel compounds [1,2,3,4].

Compounding this problem, clinical antibiotic resistance typically emerges within ten years of an antibiotic’s first use [5,6,7,8,9]. While antibiotic resistance is often viewed as a modern problem, resistance mechanisms are diverse and widespread with some tracing back millions of years [10,11,12,13]. In 2019, the Centers for Disease Control and Prevention (CDC) highlighted the seriousness of this problem in their report “Antibiotic Resistance Threats in the United States”, which identified several serious bacterial threats for which treatments are severely limited. Among the most urgent threats are Carbapenem-resistant (CRE) and Extended-Spectrum Beta-Lactamase (ESBL)-producing *Enterobacteriaceae* and various drug-resistant *Staphylococcus* and *Pseudomonas* [5,14].

*Klebsiella pneumoniae* is of particular concern with the World Health Organization classifying carbapenem-resistant *Klebsiella* as a “critical priority” [14]. Alarmingly, antibiotic resistance genes have been transferred laterally in clinics, facilitating a greater threat to patients [15]. *Klebsiella oxytoca*, while less known, has emerged as clinically relevant in recent years, primarily in immunocompromised patients and neonatal intensive care units [16,17]. These challenges underscore the urgent need for novel treatment strategies.

Bacteriophages (phages), viruses that infect bacteria, may offer one such alternative. Phages are the most abundant biological entity on the planet [18,19]. Their sheer numbers combined with their ability to lyse and kill their hosts makes them an attractive alternative to antibiotics [20,21,22,23,24,25,26]. Unlike broad-spectrum antibiotics, which disrupt healthy microbiota, phages are often specific to the species or even strain that they infect. However, this specificity comes at a cost in that phage therapies must be tailored to the specific target strain(s). In addition, phages must be properly characterized to avoid the transduction of virulence factors such as toxins or antibiotic resistance as well as to guard against bacterial phage resistance. Multi-phage cocktails—designed to exploit different infection routes and counter multiple resistance mechanisms—may mitigate these risks. Furthermore, advances in phage genomics and phage–host interaction studies could enable the genetic engineering of phages and/or a more rational cocktail design [27].

Herein, we report the characterization of five bacteriophages (ValerieMcCarty01–ValerieMcCarty05) active against a clinical *K. oxytoca* strain. Genomic and proteomic analysis revealed high sequence identity (~95%) across the phages with notable variability in one of their tail fiber genes that may explain observed differences in their host range efficiency. These phages were also able to infect several *K. pneumoniae* strains in addition to their *K. oxytoca* host, and preliminary evidence suggests they display differential bacterial resistance mechanisms.

## 2. Materials and Methods

### 2.1. Bacteriophage Isolation, Sequencing and Annotation

All phages were isolated from wastewater and propagated on a clinical *K. oxytoca* isolate from a patient with a leg ulcer as previously described for phages ValerieMcCarty03 and ValerieMcCarty04 [28]. Once purified, they were grown to high titer (≥10^8^ pfu/mL) at 37 °C for 2 days. DNA isolation and sequencing was performed using the Norgen Phage DNA isolation kit (Norgen Biotek, Thorold, ON, Canada) and the Illumina TruSeq DNA Nano kit (Illumina, Inc., San Diego, CA, USA), respectively. The de novo assembly of phages ValerieMcCarty01, 02 and 05 by Geneious version 8.0.5 [29] failed, and they were subsequently mapped to Kp1 (MG751100.1), vB_KpnM_GF (MK421971.1) and JD18 (NC_028686.1), respectively. Gene annotation was completed using DNA Master version 5.22.5 [30] to predict gene calls, GeneMarkS [31] to determine coding potential, and BLASTp [32,33,34] for protein function. Gene starts were extended to favor the coding potential. All software for assembly and annotation was used at default settings. The complete genomic sequences for ValerieMcCarty03 (accession no. OR106137) and ValerieMcCarty04 (OR271596) are publicly available on the National Center for Biotechnology Information (NCBI) database. ValerieMcCarty01, 02 and 05, however, remain as incomplete genomic sequences despite multiple sequencing rounds and are listed under accession no OR354875, OR354876, and OR354874, respectively.

### 2.2. Bacterial Kill Curves in Liquid Culture

An overnight culture of the original *K. oxytoca* clinical host grown in Lysis Broth (LB) was diluted a hundred-fold, grown for one hour and then split into ten cultures and infected with ValerieMcCarty01, 02, 03, 04 and 05 each in duplicate at an MOI of approximately 1. Bacterial count was estimated using an OD_600_ of 1 as 5 × 10^8^ *Klebsiella* cells. OD_600_ time points were taken at phage addition (time zero), 60 min, 100 min and 180 min. The data was graphed in GraphPad Prism version 11.0.0 (GraphPad Software, Boston, MA, USA, www.graphpad.com), and significant differences between phage and control time points were determined by a two-tailed test.

### 2.3. Electron Microscopy

SEM grids were prepared by adding 15 µL of a high-titer lysate to a 200-mesh copper carbon type-B electron microscope grid. The lysate was carefully removed by wicking it away with a wipe, washed once with distilled water and stained with 15 µL of uranyl acetate (0.02 g/mL) for two minutes. The grids were allowed to dry prior to stain removal and imaged with an FEI Helios NATOCAB 600i DualBeam FIB/SEM (Thermo Fisher Scientific, Hillsboro, OR, USA) with a STEM detector (BYU Electron Microscopy Facility, BYU, Provo, UT, USA). Phage capsid and tail measurements were calculated using ImageJ [35] (version 1.53k) and averaged from a minimum of four separate measurements.

### 2.4. Genomic and Proteomic Comparisons

Phages with any similarities to ValerieMcCarty phages were obtained from NCBI by BLASTp [36] query of the major capsid protein, and the corresponding bacteria were downloaded from GenBank. Kalign [37] was used to obtain the average nucleotide identity (ANI), and dot plots of nucleotide and protein sequences were generated with Gepard [38]. The lower color limit and grayscale start were lowered in dot plot generation to allow for lower levels of homology to be visible. VirClust [39] was used to convert the genome into a full proteome using the bacteria, archaea, prokaryotic viruses and plant plastid code, and protein and genome clustering predictions were performed under the default settings. PCR amplification and Sanger sequencing (Department of Biology DNA Sequencing Center at Brigham Young University) were performed on the long tail fiber genes of ValerieMcCarty01, 02 and 05 to allow for accurate protein comparisons due to the ambiguities in the whole-genome sequencing. Long-tail fiber protein sequence alignments were performed on the updated sequences with NCBI COBALT [40] and colored using the conservation color scheme.

### 2.5. Phage Phylogenetic Analysis

Multiple sequence alignments were generated with MAFFT [41] using the E-INS-i method [42], which compares conserved domains and accounts for the generalized affine gap cost [43]. Neighbor-joining trees using MEGAX version 11.0.13 [44] were used with default settings, but 1000 bootstrap iterations were performed.

### 2.6. Host Range

The host range for all five ValerieMcCarty phages was obtained by the spot method and then confirmed by duplicate plaque assay. Briefly, candidate bacterial hosts were grown overnight in LB, and 0.5 mL was plated in LB top agar and allowed to solidify for 30 min. Concentrated phage lysates (≥10^8^ pfu/mL) were diluted 10-fold and spotted (5 μL) directly onto plates. The plates were incubated for 24 h at 37 °C prior to yes/no scoring. This initial test was conducted three times. Because some spot tests were difficult to score on the *Klebsiella* hosts, these negatives were further confirmed by duplicate plaque assays using undiluted high-titer lysates. Any positives were also tested using duplicate plaque assays in tandem with the original host by incubating 50 μL of diluted phage with bacteria and plating in top agar to obtain the efficiency of plating (EOP). Diluted top agar (0.35% agar) was necessary to see the plaques clearly with some hosts due to the small plaque size of these large phages as well as their faint appearance.

### 2.7. Mass Spectrometry

Overnight culture of the original host was diluted back into 2 L of fresh LB and allowed to grow until it reached an OD_600_ value that was 5–10% of its stationary phase. ValerieMcCarty03 and 04 phage lysates were added to achieve a multiplicity of infection (MOI) of 0.025 and harvested when stabilization occurred following a crash in the culture OD_600_ measurements. The phage lysate was collected and purified by spinning the culture at 12,000× *g* for 15 min at 4 °C. The collected supernatant was then treated with 1 µg/mL of DNaseI and RNase A. ValerieMcCarty03 and 04 phages were then concentrated using a centrifugation method. Briefly, the lysate was spun 7000× *g* for 18 h at 4 °C, and the phage pellet was carefully washed with gelatin-free SM buffer prior to being spun a second time at 12,000× *g* for 10 min at 4 °C to remove any remaining cell debris. Solid CsCl was slowly added, and the samples were spun at 25,000 rpm for 24 h at 5 °C to separate the phage using a CsCl gradient. The resulting phage band was extracted from the tube by drawing it out with a needle and syringe and dialyzed overnight at 4 °C in 1 L of gelatin-free SM buffer containing 1 M NaCl. Two additional rounds of dialysis were performed at room temperature in 1 L of gelatin-free SM buffer for 2–3 h each, and then the sample was filter sterilized.

Protein extraction and trypsin digestion was performed by adding five parts ice-cold acetone to 1 part of the phage suspension (*v*/*v*) and centrifuging briefly at 13,000× *g* for 5 min. The supernatant was carefully removed, and the pellet was allowed to air dry prior to being resuspended and sonicated in 200–500 µL of 50 mM ammonium bicarbonate. Trypsin digestion was performed using the FASP digestion kit (Abcam, Waltham, MA, USA), and ZipTip pipette tips (Milipore, Burlington, MA, USA) were used for sample desalting. Final sample preparations for liquid chromatography-tandem mass spectrometry (LCMS-MS) analysis were performed using Fastprep (MP BioMedicals, Irvine, CA, USA). LC-MS/MS was run at the Department of Chemistry Mass Spectrometry Facility at Brigham Young University using an EASY-nLC 1200 LC (Thermo Fisher Scientific, Waltham, MA, USA) and Orbitrap Fusion Lumos Tribrid Mass Spectrometer (Thermo Fisher Scientific, Waltham, MA, USA). Samples were run with a flow rate of 300 nL/min, and an Orbitrap Resolution of 120K. Spectra were analyzed against a 6-frame translation of the ValerieMcCarty03 and 04 nucleotide sequence (produced using EMBOSS [45]) using PEAKS DB (BioInformatics Solutions Inc., Waterloo, ON, Canada) [46], which identifies proteins by integrating database searching with de novo sequencing to provide complete peptide identification, including peptides containing diverse modifications, sequence variants, and previously uncharacterized sequences.

## 3. Results

### 3.1. Isolation of Five Myoviruses That Infect a Clinical Klebsiella Oxytoca Strain

**Figure 1:**
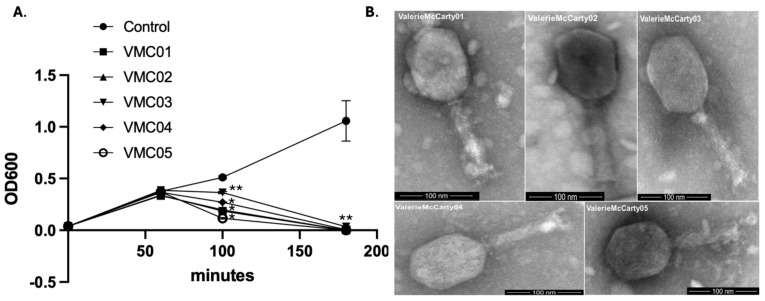
Basic phage characterization through bacterial kill curves and phage electron microscopy. (**A**) Bacterial growth in the presence of ValerieMcCarty phages (abbreviated VCM01–VCM05). Bacterial overnight was diluted a hundred-fold and the phage was added at time zero. OD_600_ is shown. Significant differences (*p*-value < 0.01 indicated by * and <0.1 indicated by **) were determined by two-tailed *t*-test. VMC01 and VMC02 data overlap in the curve shown. (**B**) Representative scanning Transmission Electron Microscope Images of *Klebsiella* bacteriophages ValerieMcCarty01, 02, 03, 04 and 05. Phage EM grids were stained with uranyl acetate and imaged with a FEI Helios NATOCAB 600i DualBeam FIB/SEM with a STEM detector.

ValerieMcCarty phages (ValerieMcCarty01-ValerieMcCarty05) were isolated from raw sewage directly against a clinical *K. oxytoca* strain obtained from a leg ulcer, as previously described [27]. All five phages could clear a culture of their host within 200 min (MOI ~1) with no significant decrease in bacterial OD600 seen until after 90 min post infection, which is likely due to their large size (Figure 1A). Full genome sequencing (Illumina NGS) and de novo genome assembly yielded circular contigs for ValerieMcCarty03 (OR106137) and ValerieMcCarty04 (OR271596) [27], but ValerieMcCarty01 (OR354875), ValerieMcCarty02 (OR354876) and ValerieMcCarty05 (OR354874) did not assemble de novo and were initially mapped to *Klebsiella* phage references to try to obtain full genomes. They remain as incomplete genomes due to unresolvable single bp ambiguities found throughout their genomes despite multiple rounds of sequencing with 100–500-fold coverage in most areas of the genome. This difficulty may be due to the highly modified DNA reported for other T4-like phages.

Both electron microscopy and genomic analysis (below) confirmed these phages have myovirus structures consistent with their classification into the *Tevenvirinae* subfamily as T4-like phages (Figure 1B). These phages have an average capsid width of 77.9 ± 4.7 (*n* = 5), capsid length of 107.3 ± 6.1 (*n* = 5), tail width of 17.9 ± 1.4 (*n* = 5), tail length of 103.1 ± 4.3 (*n* = 5), and collar of 23.8 ± 0.2 (*n* = 2, ValerieMcCarty01 and 04).

### 3.2. ValerieMcCarty Phage Genomes and Proteomes Display Genomic Similarity to Other T4-like Enterobacterales Phages

ValerieMcCarty phages display high genomic similarity with one another with the largest genome belonging to ValerieMcCarty03 with 168,196 bp and 277 ORFs (Table 1). The data in Table 1 may change slightly with complete genomic data as ValerieMcCarty01, 02 and 05 are incomplete genomes harboring single nucleotide ambiguities at 0.2% (ValerieMcCarty05)–1.3% (ValerieMcCarty01) of their genome. The genomes of ValerieMcCarty01, 03, 04, and 05 are highly variable with respect to encoded tRNAs, containing 16, 14, 17 and 18 tRNAs, respectively, with methionine tRNAs appearing multiple times within all four genomes. The lack of several tRNAs in ValerieMcCarty02 is likely due to poor sequence quality in this area (only five tRNAs were identified, and several nucleotide ambiguities are present in this area). The presence of tRNAs in phage genomes generally appears in lytic phages and is thought to provide tRNAs that are resistant to cleavage by host enzymes [47]. This may give an advantage to the phage in protein translation, especially when expanding the host range [48,49].

**Figure 2:**
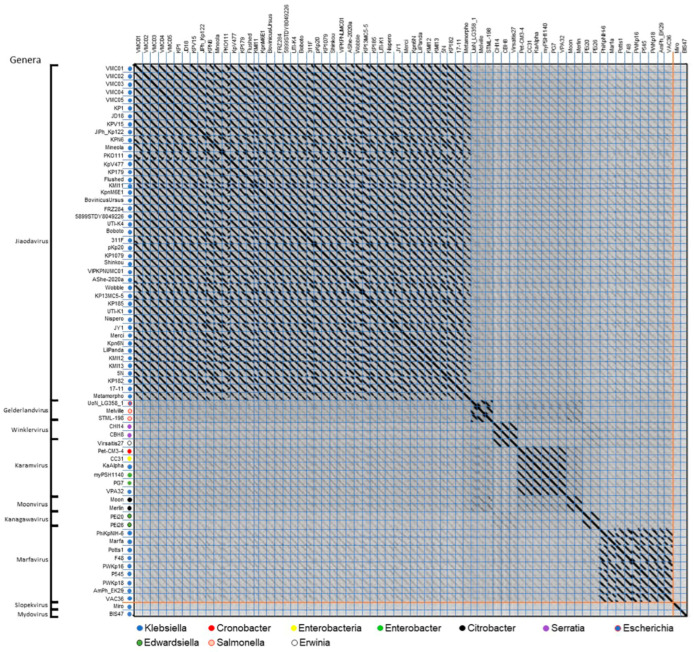
ValerieMcCarty phage genomes cluster with *Klebsiella Jiaodaviruses* and show similarity to numerous other *Tevenvirinae Enterobacteriaceae* phages available in NCBI GenBank. All phages were compared by whole-genome dot plot using Gepard [38]. Blue lines separate individual phage genomes, and orange lines indicate the start of distantly related *Klebsiella* phages that were included to highlight diversity within this group of phages. Phage host genera are indicated by colored circles according to the key at the bottom of the plot. The phage taxonomic genera obtained from NCBI Genbank are provided on the far left. Phages ValerieMcCarty01, ValerieMcCarty02 and ValerieMcCarty05 are incomplete genomes with less than 1.4% ambiguities, while ValerieMcCarty03 and ValerieMcCarty04 and other phages are complete.

Genomic relatedness to other phages was explored through BLASTn [32,33] of the *Caudovirales* nucleotide database using the ValerieMcCarty03 phage genome as the query (November 2024). From this BLASTn, 65 phages had high homology (>88.59% identity) with more than 57% of the query genome being represented. All hits were reported to infect the *Klebsiella* genus except for one (*Raoultella* phage M21a, PQ181262). Taking into consideration the mosaic nature of phage genomes, whole-genome dot plot analysis was also performed to detect homologous regions among these phages even in the presence of chromosomal rearrangements (Figure 2). All phages in Figure 2 fall within the T4-like cluster proposed by Grose and Casjens [50] and are classified as the *Straboviridae* family and *Tevenvirinae* subfamily [51] with the exceptions of two apparently unrelated *Klebsiella* phages shown at the bottom of the dot plot for comparison (phages Miro, also of the *Straboviridae* subfamily but from the *Slopekvirus* genus, and BIS47 [52] of the *Vequintavirinae* subfamily).

This dot plot-based genomic analysis suggests that the ValerieMcCarty phages belong to the ICTV-recognized *Jiaodavirus* genus [51], which is a genus of large T4-like phages with genomes 158–171 kbp. Phages that infect eight other *Enterobacterales* genera showed less but distinct genomic similarity (faint gray diagonal lines) to ValerieMcCarty phages. These phages belong to six other phage genera within the *Tevenvirinae* (*Gelderlandvirus*, *Winklevirus*, *Karamvirus*, *Moonvirus*, *Kanagawavirus*, and *Marfavirus*), highlighting the range of diversity within T4-like phages. Dot plot clusters were defined as previously outlined by Hatfull et al. as >50% genomic similarity over the length of the genome [53].

To further quantitate the degree of similarity between the ValerieMcCarty phages and the phages that infect several different *Enterobacterales* genera, the average nucleotide identity (ANI) was also calculated for a few representative phages (Table 2). When the nucleotide sequences are >95% identical, phages are classified into the same species by ICTV [54]. The two fully sequenced ValerieMcCarty phages (03 and 04) share 95.04% ANI and thus belong to a single species. The incomplete sequences of the other three also suggest they may belong to this species when fully sequenced, since they currently share >95% ANI with ValerieMcCarty03 or 04 and have 0.2–1.3% ambiguities. All *Klebsiella* T4-like phages analyzed are highly similar and belong to the *Jiaodavirus* genus, having an ANI of >92%, whereas phages known to infect other hosts have an ANI of 50–61% (excluding UoN_LG358_1 which was ~40%) and belong to other genera. The bacterial hosts represented here are known to cause significant pathology in animals and plants, suggesting these phages have coevolved and perhaps contributed to the evolution of these bacterial pathogens. Overall, this degree of genomic similarity places the ValerieMcCarty phages in a ubiquitous subfamily of successful T4-like phages, the *Tevenvirinae*, whose phages can infect multiple bacterial genera [55], making the host range of particular importance for these phages.

**Figure 3:**
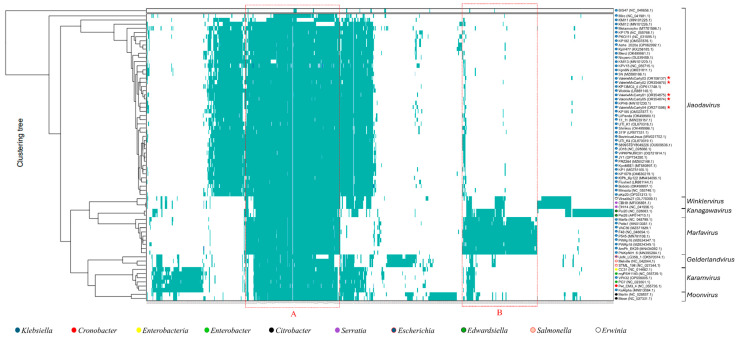
Proteomic heatmap of ValerieMcCarty phages with *Klebsiella* and *Enterobacteriaceae* phages. Proteins within each phage were identified, grouped into protein clusters and displayed as a heatmap with VirClust [39] using the pVOGs database and hhsearch. Teal vertical lines indicate a positive match to a protein cluster. The cluster of proteins shared between *Jiaodaviruses* and other *Enterobacteriaceae* phages is surrounded by a red dashed box labeled A. Box B surrounds proteins that belong to protein clusters that are unique to *Marfaviruses*. Phage genera are listed and ValerieMcCarty phages are marked with red stars (ValerieMcCarty01, 02 and 05 are incomplete genomes). Phage hosts are distinguished by colored circles preceding the phage name as specified in the key.

A proteomic phylogenetic analysis of ValerieMcCarty01–05 also suggests they belong to the *Jiaodavirus* clade (Figure 3). Consistent with the nucleotide data, these phages share a significant amount of homology with other *Enterobacteriaceae* phages when compared at the proteomic level (marked with a red box and labeled A). Kirsch and coworkers characterized T4-like phages as having variable accessory proteins and two blocks of 24 core syntenic replication and structural genes [55,58,59]. When comparing to other *Jiaodavirus* phages, CoreGenes 5.0 [60] analysis of representative phage ValerieMcCarty03 also shows 259–265 gene products are shared. The composition of the other clades within the tree mirrors the clusters observed in the genomic dot plot analysis (see Figure 2).

In Figure 3, one can also observe the conservation of Box A with phages from the six other T4-like genera. These phages show >63% proteome conservation. For example, when compared with representative phage ValerieMcCarty03, CoreGenes 5.0 [60] identifies 182 (66% of the proteome) gene products in common with *Winklervirus Serratia* phage CBH9 (MF036691), 174 (63%) in common with *Marfavirus Klebsiella* phage F48 (NC_048694) [61], 166 (60%) gene products in common with *Kanagavirus Edwardsiella* phage Pei20 (NC_028683), 180 (65%) in common with *Gelderlandvirus Salmonella* phage *Melville* (NC_042044) [62], 192 (69%) in common with *Karamvirus Enterobacter* phage PG7 (NC_023561.1), and 193 (70%) in common with *Moonvirus Citrobacter* phage Moon (NC_027331) [63]. In each case, these proteins include DNA replication, recombination and repair genes and major structural proteins. These results are consistent with the previous classification of these phage genera as belonging to the Tevenvirinae subfamily [51] and T4-like phage cluster described by Grose and Casjens [55]. Each genus also harbors proteins unique to itself. For example, the *Marfavirus* genus has a large grouping of protein clusters (indicated by a red box labeled B), although some of the proteins within this cluster are present in other phages.

*Klebsiella* phage Miro [64] and *Vequintavirinae* phage BIS47 [52] (top 2 phages in Figure 3) branched off early from the ancestral phage, giving rise to other *Klebsiella* phages. This explains the lack of genomic similarity these phages have with the T4-like *Klebsiella* phages (the *Jiaodaviruses*). However, Miro and BIS47 still share 111 and 16 proteins, respectively, with representative phage ValerieMcCarty03 as determined by CoreGenes5.0 [60]. The shared proteins with Miro (which is also from the Straboviridae family but of the Slopekvirus not Tevenvirinae subfamily) include 43 structural and assembly genes, 38 DNA replication, recombination, repair and nucleotide metabolism genes, 5 cell lysis genes, 7 transcriptional regulators, 15 hypotheticals and 3 others, suggesting a common ancestry and lifestyle. In contrast, the 16 shared proteins with phage BIS47 include 9 hypothetical, 5 DNA replication, recombination, repair and nucleotide metabolism genes, and 2 cell lysis genes (note the absence of structural genes).

ValerieMcCarty phage structural proteins were further analyzed to give insight into the evolutionary history of these phages. When the major capsid proteins (MCPs) were analyzed, all ValerieMcCarty MCP sequences were identical except for ValerieMcCarty02, which differs by seven amino acids despite good sequencing fold coverage in this area. These MCPs shared 83% similarity to other T4 MCPs by BLASTp analysis. All five phage MCPs were also closely related to those found in other *Klebsiella* phages, *Klebsiella* bacterial genomes, and other *Enterobacteriaceae* with >96%, >98%, and >82% identity, respectively. The prevalence of the phage MCP-encoding genes within bacterial genomes may suggest these phages incorporate their DNA into the host genome [50] by undergoing a lysogenic life cycle under unknown conditions; however, we were unable to find evidence of lysogeny in the lab by plaque assay. Nor did we find integrases when annotating their genomes. The large terminases of the VMC phages are almost identical except for ValerieMcCarty02 and ValerieMcCarty03, which have two adjacent amino acids that are the same but unique from the other ValerieMcCarty phages. When compared against the T4 terminase, the ValerieMcCarty phages share 83% identity. These findings suggest that ValerieMcCarty phages utilize a headful packaging strategy [65,66,67], which is consistent with the circularization we observed when assembling the genomes of ValerieMcCarty03 and ValerieMcCarty04 de novo.

### 3.3. Mass Spectrometry Suggests Capsid-Associated Enzymes, Supports 40 Proteins as Virion Components and Suggests 39Hypothetical Proteins as Proteins

To further characterize these phages and better understand their lifecycle, mass spectrometry was performed on CsCl purified ValerieMcCarty03 and 04 phages to determine proteins comprising the virion. As predicted, the presence of phage structural, DNA-associated, and host control proteins was detected in both phages (Table 3). The number of peptide spectrum matches to individual proteins were remarkably similar for both phages, which is consistent with their genomic similarity. A total of 153 and 135 proteins were detected from ValerieMcCarty03 and 04, respectively, with at least two peptide spectrum matches. The 40 proteins with >15 unique peptide spectrum matches are shown in Table 3 with complete results available in Supplementary Data S1 and S2. For Table 3, the cutoff of 15 was chosen due to the natural cutoff of known virion proteins at this point, whereas proteins detected below this may be due to contaminates that came through the virion purification process. These data suggests at least 40 proteins comprise the mature virion on these phages.

In addition to phage structural proteins, phage DNA-associated and host control proteins were also detected with >15 peptide spectrum matches. The presence of phage DNA-associated proteins is important for preventing the phage DNA from being degraded by host defense systems like CRISPR-Cas9 during injection into the cell [68], for aid in the circularization of the linear phage genome [69] and for supplying the regulatory proteins needed for phage transcription and host takeover [70]. The detection of an ADP-ribosyltransferase has been shown to regulate the transcription of host DNA to phage DNA through the ADP-ribosylation of host proteins (such as RNA polymerase) [71] and may provide these phages with an immediate advantage when entering the cell. Other modifications can also aid in the transition from early-phage gene transcription to the transcription of middle and late-phage genes.

Despite being relatives of well-characterized T4-family phages, ValerieMcCarty phages have large genomes that encode for many conserved hypothetical proteins of unknown function. Representative phage ValerieMcCarty03 harbors 94 hypothetical proteins (34% of its predicted proteome) and several other proteins with unknown function that contain functional domains (such as zinc-finger or DUF containing proteins). Analysis of the hypothetical proteins reveals 36.5% of gene products have no known function but do have a BLASTp hit, while 0.1% are considered novel when using an E-value of 1 × 10^−7^ as a cutoff. Although the mass spectrometry approach taken was to identify structural components in purified virions, mass spectrometry analysis also suggested the translation of a quarter of previously annotated hypothetical proteins in ValerieMcCarty03 (24/94) and ValerieMcCarty04 (22/96). (The ratios provided are hypotheticals with at least two peptide spectrum counts/total hypotheticals.) If spectral counts for the two phages are combined for protein homologs (BLASTp matches with e-value less then E-25), 33 hypotheticals were identified for both ValerieMcCarty03 and ValerieMcCarty04. Of those, 27 are found in both phages, leaving six uniquely identified in each phage (39 total hypotheticals). All hypothetical proteins contained low peptide counts, suggesting they are likely not abundant virion components but rather may be in fact expressed during infection and are contaminating the samples (see Supplementary Data S1 and S2).

When analyzing peptide spectra data, a total of 119 spectra for ValerieMcCarty03 and 91 from ValerieMcCarty04 did not correspond with the predicted protein sequences of these phages. However, analysis of these spectral data by tBLASTn revealed 74 of the ValerieMcCarty03 spectra and 54 of the ValerieMcCarty04 spectra matched to the unannotated portions of the genomes (see Supplementary Table S1 and Supplementary Data S1 and S2 “NA” tab). The remaining 44 and 36 spectra (less than 1% of total spectra), respectively, had no BLASTp hit, suggesting ~40% of the unmatched spectra are background spectra. The unannotated peptide spectrum matches that correspond to the wrong reading frame of a gene, an alternate strand, or landed in an intergenomic region for ValerieMcCarty03 were 64, 4, 6, respectively, and 49, 0, and 3 for ValerieMcCarty04. The peptides from both phages mapped to a total of 80 regions of which 22 are conserved in both phages. These peptides require a further, focused study to verify their expression and determine if they are nonfunctional/spurious or purposeful peptides.

### 3.4. Multiple Tail Fibers, Lysozymes, and Endolysins Likely Contribute to Lytic Activity

Phage tail fibers play an important role in host recognition and may be encoded as fusion proteins with enzymatic domains such as lysozymes and endolysins that are involved in breaking down the cell wall. In total, four tail fibers were identified in the ValerieMcCarty phages: long, short and two tail lysozymes. The short tail fiber is an adhesin responsible for binding LPS or another cell surface marker to secure the phage in place [72,73]. When the sequences of the ValerieMcCarty short tail fibers were analyzed with BLASTp, *Klebsiella* phages Mineola [74], FRZ284, JD18, K65PH164, and VIPKPNUMC01 [75] had the highest homology with >99% query coverage and >97% identity followed by several other *Klebsiella* phages. These results were expected, since the ValerieMcCarty phages were isolated using a *Klebsiella* host. The closest non-*Klebsiella* match for the short tail fiber was from *Raoultella* phage M21a (99.13% identity) and *Erwinia* phage Cronus (Query Coverage: 98% and % Identity: 46%). Interestingly, *Enterobacter* (CC31, PG7, myPSH1140 [76] and more), *Cronobacter* (Pet-CM3-4 [77])*, Serratia* (CHI14), *Kosakonia* (Kc304 [78]), *Citrobacter* (CkP1 [79]) and *Edwardsiella* (PEi26) phages matched with at least 70% identity according to BLASTp analysis with the first tail lysozyme. The same general group of phages were also observed to have >70% identity with the second tail lysozyme, but six *Salmonella* phages additionally showed homology with lysozyme 2 (*Salmonella* phage PSE-D1 [80] had both a query coverage and % identity > 99% with lysozyme 2).

**Figure 4:**
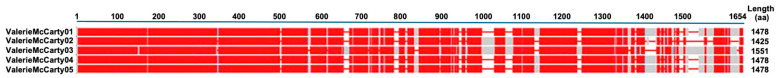
Comparison of the long tail fibers of the ValerieMcCarty01–05 Phages. Red coloration represents conserved regions, whereas gray indicates differences in at least one amino acid. Gaps in one protein are caused by unique regions found in another tail fiber.

The long tail fiber of a phage can have varying degrees of diversity and plays a role in both host attachment and host range. The N-terminal region is generally more conserved and is known to encode for the basic architecture for the tail fiber, whereas the C-terminal region is involved in binding to the phage receptor. In contrast to the MCP and the large terminase-encoding genes harboring >99% identity, the long tail fibers of the ValerieMcCarty phages displayed various levels of diversity, and their sequences were verified by Sanger sequencing to ensure accuracy (Figure 4). Conserved regions within the tail fibers are colored red, residues not found in all five proteins are colored gray and gaps represent missing residues.

The long tail fibers of ValerieMcCarty01, 04 and 05 are >99.7% identical and only differ by up to three residues. Comparison of these conserved tail fibers (represented by ValerieMcCarty04) highlight the diversity of ValerieMcCarty02 (Query Cover 87%, % Identity 75.35) and 03 (Query Cover 100%, % Identity 52.78). BLASTp of ValerieMcCarty04 gp262 (long tail fiber) revealed the 66 top hits (all which had 100% query coverage) to be *Klebsiella* phage K65PH164 followed by several other *Klebsiella* phages as the most closely related phages and then a *Raoultella* phage M21a (proteins XEM82598 and XEO41140) and a *Salmonella* phage PSE-D1 (95.78% identical, USL87069). Meanwhile, the top BLASTp hits for the long tail fibers of ValerieMcCarty02 and ValerieMcCarty03 were *Klebsiella* phage phiKP_22 (97.81% and 99.45%, respectively) followed by many of the same hits but with different percent identities. The differences in long tail fiber protein similarity, with percent identity as low as 52.78% compared to the high identity for other structural proteins such as the MCP, may allow these phages to have unique host ranges by gaining the ability to bind to unique surface receptors among various *Klebsiella* strains; however, other proteins may also contribute to their host range.

### 3.5. Host Range of ValerieMcCarty Phages Encompasses Several Clinical Klebsiella Strains and Enterobacteriaceae

**Figure 5:**
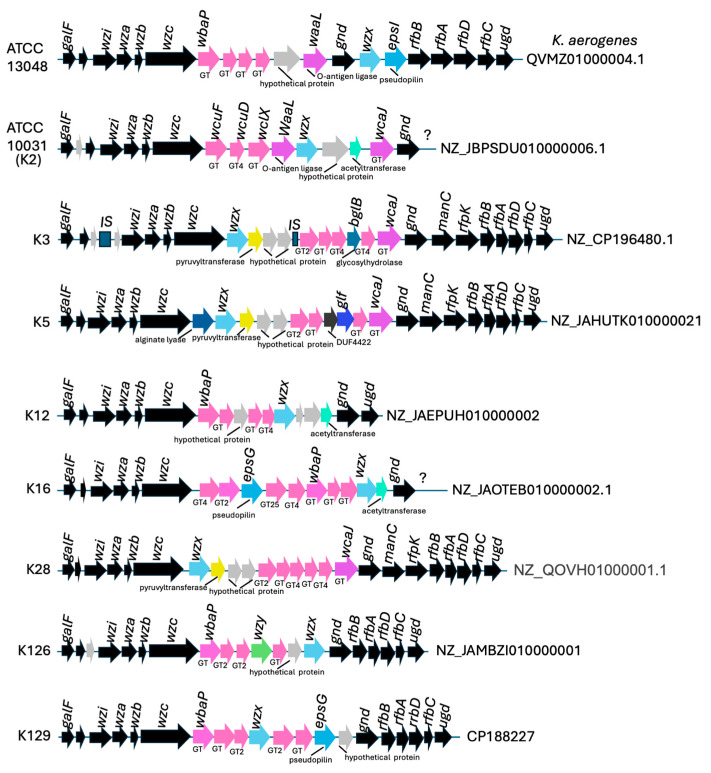
Diagram representing the capsule polysaccharide synthesis (cps) region of select *Klebsiella* strains used for host range study. The NCBI GenBank accession number from which the DNA regions and associated annotations were derived is provided on the right of each diagram. A question mark means the sequence was incomplete and the rest of the region was unknown. GT stands for glycosyltransferase with numbers given with the associated GT protein family if reported, IS are identified transposase elements, rfb genes are involved in LPS D-galactin polysaccharide and the *wz* genes (*wzi*, *wza*, *wzb*, *wzx*) are responsible for the export, assembly and surface retention of the capsule, while the *wzy* polymerase elongates the LPS chain. Annotated proteins in the middle variable regions are colored with proteins of similar function colored similarly.

Considering the range of diversity within the tail fibers of ValerieMcCarty phages, we investigated the breadth of their host range by testing phage susceptibility to a variety of *Klebsiella* species (*K. pneumoniae*, *K. oxytoca* and *K. aerogenes*) and other related genera. Host range experiments were performed in parallel against 16 antibiotic-resistant *Klebsiella* clinical isolates (14 *K. pneumoniae* and 2 *K. oxytoca* including the original host) made available from the CDC and local hospitals, non-antibiotic-resistant (non-AR) *K*. *pneumoniae* (ATCC 10031) and *K*. *aerogenes* (ATCC 13048), and a few other non-AR *Enterobacteriaceae* strains. Clinical isolates were included to further evaluate the potential of VMC phages for clinical use and represent many diverse resistance mechanisms (see Supplementary Table S2) and diverse capsular types (Figure 5).

The capsular (K antigen) type is a key determinant of *Klebsiella* virulence [81,82] and can affect infection severity as well as comorbidities [83]. For example, K3 was shown to cause rhinoscleroma [84], while K5, K12, and K28 have been associated with severe infections of the circulatory system [85], the nervous system, and the digestive system [83], respectively. Capsular type can be determined by sequencing the genes involved in capsule polysaccharide synthesis (cps), which generally extend from *galF* to *ugd.* The 5′ (encoding *galF*, *wzi*, *wza*, *wzb* and *wzc*) and 3′ end (from *gnd* or *ugd*) of the cps region is often well conserved with the variable middle region encoding for proteins involved in polymerization and assembly (GTs, wzx flippase, wzy polymerase, and modifying enzymes) primarily contributing to K-type variability [86]. Examples of the cps region from *Klebsiella* strains used in this paper are shown in Figure 5, with their corresponding NCBI GenBank accession numbers indicated on the right. Each of these strains harbors a uniquely sequenced and annotated cps region, suggesting differences in K-type and phage sensitivity.

When measured on the original *K. oxytoca* clinical isolate, ValerieMcCarty01–05 produced a titer > 10^9^ pfu/mL for all phages, and this titer was used to compare the efficiency of plating (EOP) to all other strains (Table 4). All five phages produced a mean EOP > 1.000 on *K. pneumoniae* (ATCC 10031, which carries the K2 capsular type) as well as *K. aerogenes* (ATCC 13048) despite their unique capsules.

Lytic activity on the clinical isolate strains was distinctly lower (near EOP 0.001–0.099) for most phage–isolate combinations. *K. pneumoniae* isolates IHC3 (carbapenemase producing), *K. pneumoniae* 1002002 (imipenem resistant from IMP4), K3, K5, K12, K16, K129, K142, and *K. oxytoca* K28 were susceptible to all five phages although with different efficiency. Interestingly, some variability in EOP is seen with several of the isolates; in particular, strains 1002002 and K16 displayed high variability in titer with phages ValerieMcCarty03 and ValerieMcCarty05 showing high EOP (see Supplementary Table S3 for detailed host range titers). No infectivity was detected on any other *K*. *pneumoniae* clinical isolates.

The high specificity for *K. pneumoniae*, *K. oxytoca* and *K. aerogenes* hosts correlates with the in silico analysis of the long tail fibers and the unique clustering of the *Klebsiella* phages by genomic analysis (Figure 2). All bacteria tested outside of the *Klebsiella* genus were resistant to the ValerieMcCarty phages, including *Salmonella enterica serovar Typhimurium*, highlighting the high specificity of ValerieMcCarty phages and its potential candidacy for future phage therapies.

Due to their high genomic similarity, a preliminary analysis of bacterial resistance mechanisms against these phages was performed. Various bacteria growing in the center of a clear plaque on the original bacterial host were isolated. Once purified, all the ValerieMcCarty phages were spotted onto the mutant to confirm resistance to the original phage and characterize any resistance gained against the other phages. For example, one phage-resistant mutant isolated from infection with ValerieMcCarty05 was also resistant against ValerieMcCarty01 and 02 infection but remained susceptible to ValerieMcCarty03 and 04 (Supplementary Figure S1). These differences in resistance to ValerieMcCarty phages were seen for several bacterial isolates. The difference in the long tail fiber of ValerieMcCarty03 could be a contributing factor, but ValerieMcCarty01, 04 and 05 had nearly identical tail fibers. This finding proposes the ability of these phages to overcome various phage resistance mechanisms and suggests very similar phages can have different bacterial resistance patterns. These mechanisms will be explored in future studies.

## 4. Conclusions

Herein, we reported the isolation and characterization of five closely related phages isolated on a clinical *Klebsiella oxytoca* host that infect several antibiotic-resistant *K. pneumoniae* and *K. oxytoca* clinical isolates. Two phages were fully sequenced (ValerieMcCarty03 and 04) [28] with the remaining three incompletely sequenced with 0.2–1.3% ambiguities, which is likely due to their large genomes and the presence of DNA modifications, as is seen for other T4-like phages [68]. Genomic and proteomic analysis suggests these phages belong to the *Jiaodavirus* genus of the Tevenviridiae subfamily, sharing >92% nucleotide identity and sharing 259–265 gene products to other *Jiaodavirus* phages (see Table 2 and Figure 3).

The *Jiaodavirus* genus consists of only *Klebsiella* phages to which the ValerieMcCarty phages belong. Several have been classified as lytic, and KPV15 has been used for phage therapy [90] due to its apparent absence of toxins or integrases. The strong lytic capacity of the ValerieMcCarty, other *Jiaodavirus,* and T4-like phages suggests great potential begging further characterization.

Mass spectrometry of purified virions was used to further characterize ValerieMcCarty03 and 04 through the identification of 40 virion proteins. This analysis identified peptides from 153 and 135 expressed proteins, respectively, suggesting capsid-associated enzymes including an ADP-ribosyl transferase which may modify protein activity to shift to phage production (see Table 3). These data also support 39 hypothetical proteins as proteins of unknown function (Supplementary Tables S1 and S2). This is a foundation to build upon in understanding the uncharacterized proteins of *Jiaodavirus* T4-like *Klebsiella* phages, which is an important genus for understanding the evolution of *Klebsiella* pathogens as well as a possible therapeutic agent.

The ValerieMcCarty phages documented herein represent novel *Klebsiella* phages with variable tail fibers despite their high genomic similarity with one another. Although very similar at both their sequenced genomic (>94%) and proteomic level (their MCPs are >99% identical), ValerieMcCarty phages exhibit more diversity among their tail fibers and lytic factors (see Figure 4) with long tail fiber proteins displaying as low as 52.75% identity. This finding suggests an intriguing mechanism for tail fiber exchange that is perhaps aided by the highly successful and ubiquitous family of T4-like phages to which these phages belong. T4 phages are well known for their lytic lifestyles, and three recombination hotspots have been identified within the T4 genome [91]. The long tail fibers of ValerieMcCarty01, 04 and 05 are nearly identical but still demonstrated some differences in efficiency of infection (Table 4). All five ValerieMcCarty phages (originally isolated on a clinical *Klebsiella oxytoca* host) were tested against 16 clinical *Klebsiella* strains. They displayed EOP values between 0.100 and 1.000 on five *Klebsiella* clinical isolates (IHC3, K5, K28, K129, and K142) and lower EOP values on four others (1002002, K5, K12, K16) with some phages displaying large EOP differences compared to the others on strains 100202, K12 and K16. These differences in EOP may be due to the contributions of additional tail fibers or other host-specific proteins (lysins, tRNAs, etc.) encoded within their genomes. In addition, the preliminary study herein suggests ValerieMcCarty phages select for different host resistance mechanisms, which may increase their effectiveness as a cocktail for phage therapy and should be studied further.

As members of the T4-like Tevenviridae phages which have a wide range of reported hosts, the ValerieMcCarty phages have a narrow specificity for *Klebsiella* hosts consistent with the *Jiaodavirus* genus to which they belong. Their observed tail fiber variability may enable them to adapt to evolving *Klebsiella* hosts, contributing to *Klebsiella* evolution. In addition, their close relationship to other well-characterized and therapeutic phages suggests these phages as potential candidates in clinical *K. pneumoniae*, *K. oxytoca* or *K. aerogenes* cases, and solicits their further characterization.

## Figures and Tables

**Table 1:** Estimated genome size, predicted ORFs, and tRNAs of ValerieMcCarty phages. Phages ValerieMcCarty01, ValerieMcCarty02 and ValerieMcCarty05 are incomplete genomes with ambiguities, while ValerieMcCarty03 and ValerieMcCarty04 are complete.

Name	Accession No.	Genome Size	ORFs	Predicted tRNAs	Ambiguities
**ValerieMcCarty01**	OR354875	166,582 bp	279	Arg, Asn, Asp, Gln, Gly, His, Ile, Lys, Met(x3), Pro, Ser, Thr, Trp, Tyr	2332
**ValerieMcCarty02**	OR354876	162,087 bp	274	poor sequence quality region	935
**ValerieMcCarty03**	OR106137	168,196 bp	277	Arg, Asn, Asp, Gln, Gly, His, Ile, Leu, Met(x2), Pro, Ser, Thr, Trp	0
**ValerieMcCarty04**	OR271596	166,087 bp	277	Arg, Asn, Asp, Gln, Gly, His, Ile, Leu, Lys, Met(x2), Pro, Ser(x2), Thr, Trp, Tyr	0
**ValerieMcCarty05**	OR354874	165,875 bp	276	Arg, Asn, Asp, Gln, Gly, His, Ile, Leu, Lys, Met(x3), Pro, Ser, Thr, Trp, Tyr, Unknown	319

**Table 2:** Average nucleotide identity comparisons of ValerieMcCarty phages and several representative *Enterobacterales* phages from NCBI GenBank also shown in Figure 2. Phages ValerieMcCarty01 (VMC01), ValerieMcCarty02 (VMC02) and ValerieMcCarty05 (VMC05) are incomplete genomes (with 0.2–1.3% ambiguities), while ValerieMcCarty03 (VCM03) and ValerieMcCarty04 (VMC04) and other phages are reported complete.

Accession No.	Hosts	Phage	Genus	VMC01	VMC02	VMC03	VMC04	VMC05
OR354875	*Klebsiella oxytoca*	VMC01	*Jiaodavirus*	100.00%	---	---	---	---
OR354876	*Klebsiella oxytoca*	VMC02	*Jiaodavirus*	94.61%	100.00%	---	---	---
OR106137	*Klebsiella oxytoca*	VMC03	*Jiaodavirus*	94.54%	95.07%	100.00%	---	---
OR271596	*Klebsiella oxytoca*	VMC04	*Jiaodavirus*	96.76%	94.28%	95.04%	100.00%	---
OR354874	*Klebsiella oxytoca*	VMC05	*Jiaodavirus*	97.07%	94.50%	94.96%	99.31%	100.00%
MG751100.1	*Klebsiella pneumoniae*	KP1	*Jiaodavirus*	93.78%	95.27%	94.99%	93.41%	93.65%
MN434095.1	*Klebsiella pneumoniae*	JIPh_Kp122	*Jiaodavirus*	93.46%	93.87%	93.41%	92.84%	93.04%
NC_055726.1	*Cronobacter malonaticus*	Pet-CM3-4	*Karamvirus*	60.12%	60.23%	60.41%	60.54%	60.37%
NC_014662.1	*Enterobacteria* (*E. coli*)	CC31	*Karamvirus*	60.19%	60.22%	60.44%	60.68%	60.49%
NC_055739.1	*Enterobacter cloacae*	myPSH1140	*Karamvirus*	59.82%	59.95%	60.01%	60.21%	60.07%
NC_027331.1	*Citrobacter freundii*	Moon	*Moonvirus*	60.42%	60.82%	60.70%	60.91%	60.76%
OK570374.1	*Escherichia*	UoN_LG358_1	*Gelderlandvirus*	40.32%	40.35%	40.58%	40.74%	40.74%
NC_042044.1	*Salmonella enterica serovar Newport*	Melville	*Gelderlandvirus*	53.96%	54.09%	54.17%	54.46%	54.42%
NC_028683.1	*Edwardsiella ictaluri*	PEi20	*Kanagawavirus*	50.83%	51.25%	51.19%	51.39%	51.19%
NC_041996.1	*Serratia* sp. ATCC 39006	CHI14	*Winklervirus*	54.79%	55.10%	55.10%	55.35%	55.20%
OL770309.1	*Erwinia rhapontici*	Virsaitis27	*Winklervirus*	54.82%	55.14%	55.10%	55.33%	55.18%

Average nucleotide identity values were determined by Kalign [56,57] and are color coordinated as follows: White: 100%, Green: >95%, Blue: >90%, Yellow: >50%, Red: <50%, ---: Repetitive comparison.

**Table 3:** Mass spectrometry of ValerieMcCarty03 and ValerieMcCarty04 (VMC03/04) purified virions confirms structural, DNA metabolism, lysis proteins, and hypothetical proteins with 40 high confidence proteins shown having >15 peptide spectra matches (PSM) in at least one of the two phages.

Gene Product (VMC03/04)	Phage Structural Proteins	# PSM Retrieved (VMC03/04)
gp195/192	Major capsid protein	1126/1158
gp264/262	Long tail fiber, proximal subunit	399/388
gp267/265	L-shaped tail fiber protein	392/373
gp182/179	Fibritin neck whisker protein	347/315
gp196/193	Capsid vertex protein	211/209
gp202/199	Highly immunogenic Hoc-like outer capsid protein	208/146
gp188/185	Tail sheath	179/218
gp179/176	Baseplate wedge subunit and tail pin	168/178
gp181/178	Tail collar fiber protein	147/110
gp175/172	Baseplate wedge subunit	143/139
gp176/173	Baseplate wedge initiator	130/140
gp190/187	Portal protein	120/112
gp178/175	Baseplate wedge tail fiber protein connector	114/96
gp189/186	Tail tube protein	98/106
gp213/210	Baseplate hub subunit and tail length	86/76
gp266/264	Hinge connector of long tail fiber distal connector	82/44
gp177/174	Baseplate wedge subunit	67/69
gp127/119	Internal virion protein	66/66
gp265/263	Hinge connector of long tail fiber, proximal	66/65
gp172/169	Baseplate hub subunit and tail lysozyme protein	52/45
gp183/180	Head–tail adaptor, head completion protein	46/51
gp214/211	Baseplate tail tube cap	44/40
gp20/19	Virion capsid and scaffold protein	43/52
gp168/165	Tail completion and sheath stabilizer protein	41/41
gp211/208	Baseplate hub subunit	37/40
gp215/212	Baseplate tail tube initiator	37/39
gp185/182	Tail sheath stabilizer and completion protein	36/41
gp180/177	Baseplate wedge subunit and tail pin	35/53
gp184/181	Neck protein	35/46
gp171/168	Baseplate wedge subunit	25/24
gp193/190	Prohead core scaffolding protein and protease	18/17
gp208/205	Baseplate wedge subunit	17/13
gp128/120	baseplate hub + tail chain A lysozyme	17/17
gp126/118	Internal virion protein	1/26
gp268/266	Distal tail fiber assembly catalyst	2/19
**Gene Product**	**Phage DNA-Associated Proteins**	**# Retrieved**
gp108/100	Starvation-inducible transcriptional regulator	26/27
gp169/166	DNA end protector protein	20/17
gp165/162	RNA ligase	18/17
gp205/202	DNA helicase	8/17
**Gene Product**	**Host Control/Metabolism**	**# Retrieved**
gp217/214	ADP-ribosyltransferase	189/191

The phage gene product number is given for ValerieMcCarty03/ValerieMcCarty04 (VMC03/04) along with the number of spectra detected (number of peptide spectrum match, # PSM) for each ValerieMcCarty03/ValerieMcCarty04 gene product (VMC03/04).

**Table 4:** Host range of ValerieMcCarty01–05(VMC01-VMC05) *Klebsiella* phages with mean observed efficiency of plating (EOP) values given. ^†^ Links and references are provided for strain details when available.

Strains	VMC01	VMC02	VMC03	VMC04	VMC05
	Mean EOP ^†^	Mean EOP ^†^	Mean EOP ^†^	Mean EOP ^†^	Mean EOP ^†^
*K. pneumoniae* ATCC 10031	1.09	1.83	8.00	1.45	3.02
*K. aerogenes* ATCC 13048	1.16	1.06	15.53	3.17	1.04 *
*K. pneumoniae* 1002002 [87]	0.001 *	1.56 × 10^−8^	0.001	0.001 *	0.72
*K. pneumoniae* IHC3	0.26	0.21	1.05	0.13	0.57
*K. pneumoniae* 1300761	0.00	0.00	0.00	0.00	0.00
*K. pneumoniae* K3 [84]	2.93 × 10^−7^ *	8.72 × 10^−7^	3.02 × 10^−7^ *	2.01 × 10^−5^	1.19 × 10^−6^ *
*K. pneumoniae* K5 [85]	0.048	0.119	0.051	0.039	0.068
*K. pneumoniae* K12 [88]	2.24 × 10^−7^ *	5.47 × 10^−7^	3.76 × 10^−7^ *	1.07 × 10^−5^	1.43 × 10^−7^ *
*K. pneumoniae* K16 [88]	2.88 × 10^−5^	0.002	0.700	4.33 × 10^−8^	4.93 × 10^−8^
*K. oxytoca* K28 [88]	0.62	1.23	0.31	0.41	0.27
*K. pneumoniae* K112 [83]	0.00	0.00	0.00	0.00	0.00
*K. pneumoniae* K126 [89]	0.00	0.00	0.00	0.00	0.00
*K. pneumoniae* K129 [83]	4.04 × 10^−4^ *	2.84 × 10^−4^ *	3.47 × 10^−4^	1.46 × 10^−4^ *	0.002
*K. pneumoniae* K142	0.001	0.001	0.002	0.001	0.002
*K. pneumoniae* K143	0.00	0.00	0.00	0.00	0.00
*K. pneumoniae* K148	0.00	0.00	0.00	0.00	0.00
*K. pneumoniae* K160	0.00	0.00	0.00	0.00	0.00
*S. enterica Typhimurium*	0.00	0.00	0.00	0.00	0.00
*E. coli* E1	0.00	0.00	0.00	0.00	0.00
*E. coli* E13	0.00	0.00	0.00	0.00	0.00
*E. coli* E114	0.00	0.00	0.00	0.00	0.00
*E. coli* E150	0.00	0.00	0.00	0.00	0.00
*E. coli* E162	0.00	0.00	0.00	0.00	0.00
*S. marcescens* S121	0.00	0.00	0.00	0.00	0.00
*S. marcescens* S122	0.00	0.00	0.00	0.00	0.00
*S. marcescens* S123	0.00	0.00	0.00	0.00	0.00
*S. marcescens* ATCC 27143	0.00	0.00	0.00	0.00	0.00
*E. coli* K12	0.00	0.00	0.00	0.00	0.00
*E. cloacea* ATCC 13047	0.00	0.00	0.00	0.00	0.00
*C. sakazakii* ATCC 29544	0.00	0.00	0.00	0.00	0.00
*Y. enterolyticus* ATCC 23715	0.00	0.00	0.00	0.00	0.00

^†^ EOP is titer on the test strain divided by the titer on the reference strain (the original ValerieMcCarty isolate). An EOP value of 0.000 means no infection was observed. Phage–host combinations that were positive for infection but had an EOP value of ≤10^−4^ are shown in scientific notation. Values are from at least two independent tests. * Plaques were faint and difficult to see and were viewed with diluted top agar for confirmation (0.35% agar).

## Data Availability

Phage genomes are available on NCBI with the following accession numbers: OR354875, OR354876, OR106137, OR271596 and OR354874.

## Supplementary Materials

The following supporting information can be downloaded at https://www.mdpi.com/article/10.3390/v18040430/s1, Supplementary Figure S1. A representative plate showing a strain that is resistant to some ValerieMcCarty Phages is sensitive to others. Supplementary Data S1. Raw mass spectrometry data and analysis of ValerieMcCarty03. Supplementary Data S2. Raw mass spectrometry data and analysis of ValerieMcCarty04. Supplementary Table S1. Peptides detected by mass spectrometry analysis of ValerieMcCarty03 and 04 that suggest frameshifts. Supplementary Table S2. Resistance mechanisms of CDC antibiotic-resistant isolates used in this study. Supplementary Table S3. Host range of ValerieMcCarty phages with standard deviations.
