# Supplementary material for: Characterization of Klebsiella Phages Isolated Against a Clinical Host with High Genome and Proteome Identity but Variable Tail Fibers
Source: Viruses. 2026 Apr 1;18(4):430. doi: 10.3390/v18040430 (PMC13119998; doi:10.3390/v18040430)
Supplement: Supplementary file 1 [file viruses-18-00430-s001.zip › Supplementary Figure S1.pdf]

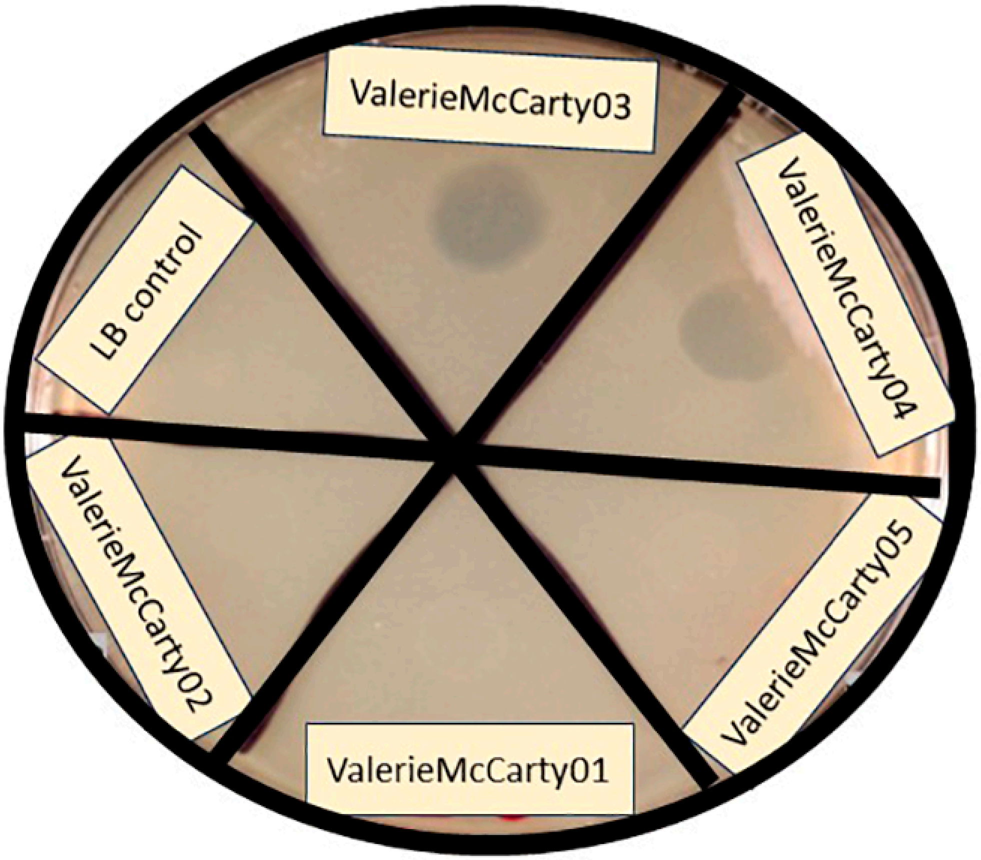

Figure S1. A Representative Plate Showing a Strain that is Resistant to Some ValerieMcCarty Phages is Sensitive to Others**.** A phage resistant mutant of *Klebsiella oxytoca* was isolated from a plate containing the ValerieMcCarty05 phage. Once purified, sensitivity to the VMC phages was reevaluated by plating an overnight culture of the phage resistant mutant in top agar and spotting 5 μL of each VMC phage high titer lysate (>10^8 pfu/ml) along with an LB control.
