# Supplementary material for: Characterization of Klebsiella Phages Isolated Against a Clinical Host with High Genome and Proteome Identity but Variable Tail Fibers
Source: Viruses. 2026 Apr 1;18(4):430. doi: 10.3390/v18040430 (PMC13119998; doi:10.3390/v18040430)
Supplement: Supplementary file 1 [file viruses-18-00430-s001.zip › Supplementary Tables.pdf]

Supplementary Table S1 Frameshift Peptides in ValerieMcCarty03 and 04

Peptide (03)	Peptide (04)	Hit in ValerieMcCarty03 (bp)	Hit in ValerieMcCarty04 (bp)	Gene Product (03)	Gene Product (04)	Function
**KALLESLTVLTKC**	RSFSGELCGPSKG	4361…4329	3909…3871	**9**	**8**	motB-like transcriptional regulator
**NVSISSSEIPWART**		4918…4956		11		exonuclease
**RISVHHAADTLRI**		7907..7866		15		anti-sigma factor protein
**XGRMNSESGAVIKS**			8089…8127	15		RNA polymerase ADP-ribosylase
**KEPCVTKIEFLLLLYTVRSTKI**		9453…9385		18		hypothetical protein
**RLALCGNHGKL**		10395..10363	10462…10494	**22**	**21**	dCTP pyrophosphatase
**KTTFSPASTKR, KDYAAIASTVSI**		10957…10986, 11659…11624		23		DNA primase
**KQNEKDCTCFSVRS**	RAGTAEQLDTAKI	12711…12670	12492…12590	**27**	**26**	spackle periplasmic protein
**KSTSSKDLLRR**	RQEAQIPRSMMITVGLKF, KIKTNGNRPIMLVMVKQ	14668…14636	13339…13395, 140655…14602	**30**	**28**	DNA primerase/helicase
**RITSSTLNQYKRL, RNDAVHQSLGTEMPSN, KQSTKTYMASIARRI**	RALSCHKKSDRK, RNTTHAFLTSRI	21285…21247, 21665…21615, 22110…22066	18622…18587, 21072…21107	**39**	**36**	DNA polymerase
**RWIIQNSWLKT**	KDGAEAPNLLLKV, RVSAASSTLLPRRT, KMLAQLSSGQQLTRA	24248…24286, 24492…24536, 24726…24682	22219…22187	**43**	**39**	DNA polymerase processivity factor
**RALFAIHGII,LIAGTELMIRM**		28350…28321, 29300…29332		48		alpha-glucosyltransferase
**KLLTVNSSLKK**		31921…31889		56		hypothetical protein
**KSSASMLTPLKV, SSASMLTPLKV**			33973…34008, 33973…34008, 33976…34008	63		hypothetical protein
**KSNFSIASHTASRPSRL**	RFSSGSLCRE	36219…36269	35326…35297	**69**	**65**	NrdD-like anaerobic ribonucleotide reductase
**KLTMSMQKSRR**			25283…25254, 25802…25770		44	recombination-related endonuclease
**KVSSANATISSSRE**			29856…29897		51	RNA polymerase sigma factor
**KSSISLVSLANGTTTM**			37463…37416		70	putative nucleotide reductase
**KSSSACKLLKNCCGKL**			37605…37558		71	antitoxin from a toxin-antitoxin system
**RSKSTWSPSGKT**		39993…40028		76		hypothetical protein
**KISSTGHSSFEIGTVSNLTSLIIINRI**		40617…40697		79		hypothetical protein
**SSISNTEFASRD**		47629…47664		96		thioredoxin
**RTVVFGSSLQRD**		48912…48880		99		hypothetical protein
**KMNSSVYLWRL, KMNSSVYLWRL**			49174…49142, 49174…49142		100	starvation-inducible transcriptional regulator
**RNTMPNLTTKF**			49629…49661		102	hypothetical protein
**RRTDSSAAKL**		50445…50416		102		hypothetical protein
**RNSLNQSAMDSGKL**		53566…53531		111		hypothetical protein
**RTSVYDKEKR,LDDFEVSFKI**	RTSVYDKEKR, TVANALELNKF	54263…54292, 54254..54292	50643…50672, 50764…50796	**114**	**106**	thymidine kinase
**KILKSSLLSLRA**	RSFLQVLNRV	57284…57249	53700…53729	**121**	**113**	RegB-like RNA endonuclease
**RCCSGVRELKR**		57592…27624		122		autonomous glycyl radical cofactor
	LLLVNSDTLLRH		63003…63038		137	Hypothetical protein
**RVALVVANRT**	RVALVVANRT	68094…68065	65670…65641	**152**	**149**	overlap with tRNA (gp149 is a tRNA)
**KSEDVLINRR**	RTQTHLQELPRS	69006…68977	66220…66255	**between 155 and 156**	**between 152 and 153**	peptide between two tRNA
**RLSSGSSKTMITVKKL**		70801…70754		164		membrane protein
**RLTDLNLVHDRN**			69467…69496		164	deoxynucleoside monophosphate kinase

**KLPEAQTYGTTLE**		71321…71362		165		RNA ligase
**PCGEPTVSVAGITPGTTAVRL**			70151…70210		165	tail completion and sheath stabilizer protein
	ISLGLIMFIWRS		72163…72128		168	baseplate wedge
**KNLSLHIMVSIHMFTQWKQ**		76175…76231		172		baseplate hub subunit and tail lysozyme
**KTSSVFVSSPRL**		78549…78584		175		baseplate wedge
**VGVSAEPNTGRR**	KDAIMADVLKL	81399…81431	77196…77164	**176**	**173**	baseplate wedge initiator
	LATTISPIKN		80215…80186		174	baseplate wedge subunit
	KVHCHKVISSLVIRA		92684…92637		185	Tail sheath
**LFASGMRERG**	KSISFTILAKK	99092…99063	95770…95802	**190**	**187**	portal protein
**RMLAANLSPGPW**		100568…100533		193		scafoolding protein
**KNPLKSLVRPVAVKV**	KWASVSINKL	102744…102788	99635….99664	**195**	**192**	MCP
**SISSGASNPLASKS**		103914…103873		196		capsid vertex protein
	TETHNLAASAGVISNALAISKS		104218…104277		198	DNA primase-helicase subunit
**KSSDTITLVQNRW**		106524…106486		between 199-200		possible peptide between genes
**KSSIISTNAVVGTMIRT**		109974…109942		204		DNA helicase
**AMLCIHRLCRFSSLQLNRI**		112821…112874		209		baseplate hub
**RNILCTLVCGQRC**		114506…114544		211		baseplate hub subunit
**KSSQLVIMLRQNSS**	KSSQLVIMLRQNSS	116671..116712	113885…113946	**213**	**210**	baseplate hub subunit tail length
	KEIEQISTYRK		115751…115719		212	tail tube protein
**SASLTSADHSIPLKR**		119364…119408		216		hypothetical protein
**RITSASSLKLDKT,RITSASSLKLDKT**	MSLSLDSLFMGPIKL, RKPFSKAESSGRI	119811…119849, 119811…119849	117124…117080, 117403…117441	**217**	**214**	ADP-ribosyltransferase
**KSGIPTDGSHGIKR**	ISYLLLLRQRL	122572…122537	119692…119724	**219**	**216**	DNA ligase
	RSCSVLSLESNLASKV		120682…120641		217	DUF3045 domain-containing protein
	NLHTTVQLSRL		121892…121921		220	hypothetical protein
**ITMCSSALKM**		126018…125989		226		Baseplate hub subunit
**NWSKQSDGASAPTLRI**		126795…126745		between 227-228		possible peptide between proteins
**KVANSFASSRTKM**	KVANSFASSRT	127832…127870	125047…125079	**231**	**228**	lysis inhibition accessory protein
**RACSIKRASTASRK**		129744…129785		235		molybdopterin-guanine dinucleotide protein
**KSSKMNLIDKQKK**		132455…132417			242	Rz-like spanin
**RITSTISSRDNPEM**		132568…132609			243	putative inhibitor of host transcription
**KLALMAKNGLRL, KLNCFSMSSSNTLRISRA**	DISTFPLGKL, QMLNTMLPFMIMPGSSRN	135092…135057, 135716…135754	133446…133475, 133617…133564	**246**	**245**	ribonucleotide reductase
**KSSLNAAIASGRL**		138315…138353		248		DUF4326 domain containing protein
**RQNGITAVSSALSVQRS,RQNGITAVSSALSVQRS,RQNGITAVSSALSVQRS**		139083…139033, 139083…139033, 139083…139033		250		thymidyalte synthase
**KSDSTVVRSMSGH**		140069…140107		252		dihydofolate reductase
	RVPFSLSLQKN		139799…139767		between 256 and 257	possible peptide between genes
	IGDSMSDLLKQ		143012…143044		257	single strand DNA binding protein
**RVSTKSSPKA, IQAALIVPLKI**	KCEFAVGAAAATETLLEV	149538…149509, 150045…150077	148517…148461	**265**	**264**	hinge connector of long tail fiber, distal
**GSPTKSSIFSTKL**	KTPLTAASPVTQSD	153742…153701	152378…152337	**267**	**265**	L-shaped tail fiber protein

**RSAALVHSKT, SLSLCTGAISVSLILRK**			161675..161704, 161816…161869	280		DNA topoisopmerase II
	KSLGPSETHSPSRR		160149…160190		283	hypothetical protein
	RGFGPLSENKLSTE		160709…160665		284 and 285	possible peptide between genes
**FEPNAEMLLLKL**		163812…163777		287		hypothetical protein
	STGIDTTSRG		162425…162454		290	rIIB lysis inhibitor
**RQTLAILFSFVRN, KCGFHHDCDRL**	RQTLAILFSFVRN, KCGFHHDCDRH	.165916…165954, 166259…166291	163508…163546, 163851…163880	**291**	**291**	rIIA lysis inhibitor

Supplementary Table S2 Resistance Mechanisms of CDC Antibiotic Resistant Isolates

Strain	Identified Resistance Mechanisms
***K. pneumonia* K3**	aac(6')-Ib, aadA1, dfrA14, KPC-3, OmpK35, oqxA, oqxA, oqxB, OXA-9, SHV-12, strA, strB, sul2
***K. pneumonia* K5**	aac(6')-Ib, aph(3')-Ia, catA1, dfrA12, KPC-2, mph(A), OmpK35, oqxA, oqxA, oqxB, OXA-9, SHV-11, sul1, TEM-1A
***K. pneumonia* K12**	aac(6')-Ib, aph(4)-Ia, catA1, cmlA1, dfrA12, OmpK35, oqxA, oqxA, oqxB, SHV-12, sul1, sul3
***K. pneumonia* K16**	LEN16
***K. oxytoca* K28**	
***K. pneumonia* K112**	aac(6'), aph(3'), aph(4), catA1, cmlA1, dfrA12, KPC-3, mph(A), oqxA, oqxA, oqxB, sul1, sul3
***K. pneumonia* K126**	aac(6')Ib-cr, catB3, dfrA1, fosA, KPC-2, OmpK36, oqxA, oqxA, OXA-1, sul1, TEM-1B
***K. pneumonia* K129**	aac(6')-Ib, aadA2, aph(3')-Ia, catA1, dfrA12, KPC-3, mph(A), OmpK35, oqxA, oqxA, oqxB, sul1, TEM-1A
***K. pneumonia* K143**	aac(3)-IId, aac(6')-Ib, armA, ARR-3, catA1, cmlA1, CMY-4, CTX-M-15, dfrA1, fosA, mph(E), msr(E), NDM-1, oqxA, oqxA, OXA-9, strA, strB, sul2, TEM-1A
***K. pneumonia* K148**	aac(3)-IId, CMY-6, CTX-M-15, dfrA14, mph(A), NDM-1, oqxA, oqxA, OXA-1, QnrB7, rmtC, SHV-11, strA, strB, sul1, sul2, TEM-1B, tet(A)
***K. pneumonia* K160**	fosA, oqxA, oqxA, oqxB, OXA-48, SHV-11
***E. coli* E1**	aac(6')Ib-cr, aadA5, dfrA17, KPC-3, mph(A), OXA-1, sul1, tet(A)
***E. coli* E13**	aac(3)-IId, aadA2, catA1, CTX-M-14, dfrA12, mph(A), sul1, TEM-1B, tet(A)
***E. coli* E114**	aadB, cmlA1, dfrA5, KPC-3, strA, strB, sul1, sul2, TEM-1B
***E. coli* E150**	aadA5, CMY-42, dfrA17, mph(A), NDM-5, sul1, TEM-1B, tet(A)
***E. coli* E162**	CTX-M-15, erm(B), NDM-7, QnrS1, strA, strB, sul2, TEM-1B, tet(A)
***S. marcescens* S121**	SME-3
***S. marcescens* S122**	SME-3
***S. marcescens* S123**	SME-3

Supplementary Table S3 Host Range Efficiency of Plating

Strains	VMC01		VMC02		VMC03		VMC04		VMC05	
	Mean EOP	stdev	Mean EOP	stdev	Mean EOP	stdev	Mean EOP	stdev	Mean EOP	stdev
***K. pneumoniae* ATCC 10031**	1.085	3.28E-01	1.833	9.10E-01	7.996	6.31E+00	1.448	4.67E-01	3.024	1.16E+00
***E. aerogenes* ATCC 13048**	1.159	1.76E-02	1.056	6.40E-01	15.529	1.61E+01	3.166	2.81E+00	1.044	6.45E-01
***K. pneumoniae* 1002002**	0.001	9.70E-05	1.56E-08	4.01E-09	0.001	2.41E-04	0.001	8.96E-04	0.715	1.15E-01
***K. pneumoniae* IHC3**	0.255	1.16E-01	0.207	2.58E-01	1.053	3.76E-01	0.130	1.93E-01	0.569	2.61E-01
***K. pneumoniae* 1300761**	0.000	0.00E+00	0.000	0.00E+00	0.000	0.00E+00	0.000	0.00E+00	0.000	0.00E+00
***K. pneumoniae* K3**	2.93E-07	2.38E-08	8.72-07	7.19E-07	3.02E-07	1.64E-07	2.01E-05	7.24E-06	1.19E-06	3.09E-07
***K. pneumoniae* K5**	0.048	5.46E-02	0.119	1.91E-02	0.051	6.99E-02	0.039	4.61E-02	0.068	3.16E-03
***K. pneumoniae* K12**	2.24E-07	1.95E-08	5.47E-07	2.01E-07	3.76E-07	1.35E-07	1.07E-05	8.47E-06	1.43E-07	4.88E-08
***K. pneumoniae* K16**	2.88E-05	2.85E-06	0.002	1.39E-03	0.700	1.33E-01	4.33E-08	4.68E-08	4.93E-08	3.50E-08

***K. oxytoca* K28**	0.621	4.37E-01	1.234	6.79E-01	0.313	3.90E-01	0.413	2.43E-01	0.272	1.93E-01
***K. pneumoniae* K112**	0.000	0.00E+00	0.000	0.00E+00	0.000	0.00E+00	0.000	0.00E+00	0.000	0.00E+00
***K. pneumoniae* K126**	0.000	0.00E+00	0.000	0.00E+00	0.000	0.00E+00	0.000	0.00E+00	0.000	0.00E+00
***K. pneumoniae* K129**	4.02E-04	2.96E-04	2.84E-04	1.51E-04	3.47E-04	4.90E-04	1.46E-04	5.82E-05	0.002	7.77E-04
***K. pneumoniae* K142**	0.001	7.65E-04	0.001	2.38E-04	0.002	1.75E-04	0.001	4.98E-05	0.002	1.47E-03
***K. pneumoniae* K143**	0.000	0.00E+00	0.000	0.00E+00	0.000	0.00E+00	0.000	0.00E+00	0.000	0.00E+00
***K. pneumoniae* K148**	0.000	0.00E+00	0.000	0.00E+00	0.000	0.00E+00	0.000	0.00E+00	0.000	0.00E+00
***K. pneumoniae* K160**	0.000	0.00E+00	0.000	0.00E+00	0.000	0.00E+00	0.000	0.00E+00	0.000	0.00E+00
Salmonella enterica Typhimurium	(0)	0.00E+00	(0)	0.00E+00	(0)	0.00E+00	(0)	0.00E+00	(0)	0.00E+00
***E. coli* E1**	(0)	0.00E+00	(0)	0.00E+00	(0)	0.00E+00	(0)	0.00E+00	(0)	0.00E+00
***E. coli* E13**	(0)	0.00E+00	(0)	0.00E+00	(0)	0.00E+00	(0)	0.00E+00	(0)	0.00E+00
***E. coli* E114**	(0)	0.00E+00	(0)	0.00E+00	(0)	0.00E+00	(0)	0.00E+00	(0)	0.00E+00
***E. coli* E150**	(0)	0.00E+00	(0)	0.00E+00	(0)	0.00E+00	(0)	0.00E+00	(0)	0.00E+00
***E. coli* E162**	(0)	0.00E+00	(0)	0.00E+00	(0)	0.00E+00	(0)	0.00E+00	(0)	0.00E+00
***S. marcescens* S121**	(0)	0.00E+00	(0)	0.00E+00	(0)	0.00E+00	(0)	0.00E+00	(0)	0.00E+00
***S. marcescens* S122**	(0)	0.00E+00	(0)	0.00E+00	(0)	0.00E+00	(0)	0.00E+00	(0)	0.00E+00
***S. marcescens* S123**	(0)	0.00E+00	(0)	0.00E+00	(0)	0.00E+00	(0)	0.00E+00	(0)	0.00E+00
***S. marcescens* ATCC 27143**	(0)	0.00E+00	(0)	0.00E+00	(0)	0.00E+00	(0)	0.00E+00	(0)	0.00E+00
***E. coli* K12**	(0)	0.00E+00	(0)	0.00E+00	(0)	0.00E+00	(0)	0.00E+00	(0)	0.00E+00
***E. cloacae* ATCC 13047**	(0)	0.00E+00	(0)	0.00E+00	(0)	0.00E+00	(0)	0.00E+00	(0)	0.00E+00
***C. sakazakii* ATCC 29544**	(0)	0.00E+00	(0)	0.00E+00	(0)	0.00E+00	(0)	0.00E+00	(0)	0.00E+00
***Y. enterolyticus* ATCC 23715**	(0)	0.00E+00	(0)	0.00E+00	(0)	0.00E+00	(0)	0.00E+00	(0)	0.00E+00

Mean EOP values (0): Only spot test was performed
